# Plasma Exchange in Pediatric Neurology Patients: A Single-Center Experience

**DOI:** 10.7759/cureus.52691

**Published:** 2024-01-21

**Authors:** Maram A Aljezani, Faris Althubaiti, Latifah Alhamed, Abdulrahman Alharthi, Abdulaziz Alamoodi, Yousof Bakheet, Maha Badawi, Salwa Hindawi

**Affiliations:** 1 Pediatric Neurology, King Abdulaziz University Hospital (KAUH), Jeddah, SAU; 2 Pediatric Neurology, King Fahad Medical City (KFMC), Riyadh, SAU; 3 Pediatric Neurology, King Abdulaziz University, Jeddah, SAU; 4 Medicine, King Abdulaziz University, Jeddah, SAU; 5 Hematology, King Abdulaziz University, Jeddah, SAU; 6 Hematology/Blood Transfusion Services, King Abdulaziz University, Jeddah, SAU

**Keywords:** outcomes, indications, myasthenia gravis, guillain-barré syndrome, neurological disease, pediatric, apheresis, plasmapheresis, tpe, therapeutic plasma exchange

## Abstract

Background: Therapeutic plasma exchange (TPE) is a procedure involving the filtration of a patient's plasma to eliminate pathogenic components or address deficiencies. This technique finds varied indications in the pediatric age group, particularly in neuroinflammatory diseases.

Objectives: The objective of this study is to delve into our local experience with TPE, focusing on indications, outcomes, and complications among children with neurological diseases at King Abdulaziz University Hospital (KAUH) in Jeddah, Saudi Arabia.

Results: Conducted at the pediatric department of KAUH in Jeddah from November 2008 to July 2023, this retrospective cohort study examined 15 patients, revealing a notable male predominance with 12 male patients (80%) and three female patients (20%). About two-thirds of patients exhibited an average illness severity, with a Glasgow Coma Scale (GCS) score of 10.7 and an Expanded Disability Status Scale (EDSS) score of 4.8. The median length of hospital stay was 23 days, and in the pediatric intensive care unit (PICU), it was 8.5 days. Presenting symptoms included limb weakness (n = 6), loss of consciousness (n = 3), dysphagia (n = 3), photophobia (n = 1), and ascending paralysis (n = 1). The TPE was performed for Guillain-Barré syndrome (GBS) (n = 7), myasthenia gravis (MG) (n = 3), transverse myelitis (TM) (n = 2), neuromyelitis optica (NMO) (n = 2), and systemic lupus erythematosus (SLE) cerebritis (n = 1). Twelve patients were admitted to the PICU, and mechanical ventilation was required for 10 patients. In magnetic resonance imaging (MRI) findings, abnormalities were observed in 10 cases, while the remaining five either had normal results or did not undergo MRI. Most patients required five sessions of TPE (n = 7). The median age at the initiation of TPE was 13 years. Twelve patients improved with TPE treatment, while three did not. Complications observed during and following TPE included fever (n = 5), electrolyte disturbance (n = 5), hypotension (n = 3), hypocalcemia (n = 2), bradycardia (n = 2), vomiting (n = 1), tachycardia (n = 1), eye rash (n = 1), infection (n = 1), and bleeding originating from the TPE procedure site (n = 1).

Conclusion: In conclusion, our study underscores the significance of TPE as a therapeutic modality, emphasizing the imperative for ongoing research to fully exploit its potential across diverse medical contexts for enhancing patient care. Our findings, consistent with prior research, reveal plasma exchange's (PLEX's) wide-ranging applications and complications in neurological disorders.

## Introduction

Therapeutic plasma exchange (TPE) is an extracorporeal blood purification method used to treat a spectrum of illnesses, including neurological diseases such as Guillain-Barré syndrome (GBS), hematological diseases like thrombotic thrombocytopenic purpura (TTP), and rheumatological diseases such as catastrophic antiphospholipid syndrome (CAPS) [1]. This procedure involves isolating plasma from whole blood and replacing it with either plasma or albumin to eliminate aberrant plasma components [2-3]. It specifically examined the most prevalent indications, complications, and outcomes of pediatric patients undergoing TPE at King Abdulaziz University Hospital (KAUH) in Jeddah, Saudi Arabia. Our findings shed light on the clinical profiles and outcomes of TPE therapy in our study population, emphasizing the necessity for personalized treatment approaches and the rigorous management of potential complications. By removing compounds such as antibodies, immune complexes, cytokines, and endotoxins and replenishing deficient plasma components [4], TPE alleviates systemic circulation from autoantibodies and inflammatory markers. This makes it applicable to a range of neuroinflammatory disorders [5]. For example, evidence supports that the serum of GBS patients exhibiting either acute inflammatory demyelinating polyneuropathy or acute axonal neuropathy clinical forms contains antibodies targeting myelin-producing Schwann cells or gangliosides on the axolemma at the nodes of Ranvier. This targeting leads to demyelination, axonal loss, and nerve damage.

Therapeutic plasma exchange removes pathogenic factors in the serum, resulting in improvements in muscle strength, accelerated recovery, and reduced mechanical ventilation requirements [6]. A meta-analysis of six trials, which included 649 GBS patients (some ≥10 years of age), revealed the superiority of TPE over supportive care [7]. Although effective when initiated within four weeks of symptom onset, optimal efficacy was observed when started within seven days. Patients treated with TPE had a slightly higher risk of relapse in the first year but were more likely to fully recover muscular strength in one year compared to those receiving supportive care [7]. The clinical effectiveness of TPE depends on factors such as the separation method, replacement solution type, exchanged volume, and the number of sessions [7].

A recent publication by Hindawi et al. [8] provides a comprehensive summary of our center's experience with TPE, covering both adult and pediatric cases. Notably, neurologic conditions emerge as the predominant indications for TPE in our center. In this study, we specifically focus on evaluating our center's experience in managing pediatric patients with neurologic conditions. The analysis includes the characteristics of patients, outcomes, and complications among pediatric neurology patients.

## Materials and methods

We conducted a comprehensive review and assessment of 15 patients under the care of the pediatric department at KAUH in Jeddah, Saudi Arabia. All data were presented graphically using line charts and illustrated graphs.

Study design and setting

This retrospective study covers the period from November 2008 to July 2023. It involves a review of records from the blood bank's apheresis procedures to identify pediatric patients with neurological disorders who underwent TPE at KAUH in Jeddah, Saudi Arabia.

Therapeutic plasma exchange is conducted at KAUH using centrifuge-based technology. Requests for TPE undergo review by the on-call apheresis physician. Cases are evaluated in an evidence-based manner, utilizing the most recent version of the Guidelines of the American Society for Apheresis (ASFA). The exchanged volume typically ranges from 1 to 1.5 plasma volumes. Replacement fluids are determined by the apheresis physician on a case-by-case basis and in consultation with ASFA guidelines. For most neurologic conditions, the replacement fluid of choice is a combination of albumin and normal saline. In smaller patients, a blood primer is used when prompted by the TPE machine. Central venous access is mandatory for all pediatric TPE procedures.

Study population

The study includes all patients aged 18 and below who underwent TPE for neurological disorders at KAUH in Jeddah, Saudi Arabia. Exclusions comprise adult patients and pediatric cases that underwent TPE for non-neurologic indications.

Ethical approval and study procedure

Ethical approval for the study's aim, protocol, and procedures was obtained from the Unit of Biomedical Ethics Research Committee at the Faculty of Medicine, King Abdulaziz University, under reference number 355-23, on June 22, 2023. To ensure confidentiality, patient personal data were anonymized, and consent was obtained before enrolling local cases.

Data analysis

The data, collected in a Microsoft Excel sheet (Microsoft Corp., Redmond, WA), underwent summarization and analysis using IBM Statistical Package for the Social Sciences (SPSS) software version 26 (IBM Corp., Armonk, NY). Descriptive statistics employed means and associated standard deviations (SD) for continuous variables and numbers and percentages (%) for categorical variables.

## Results

**Figure 1:**
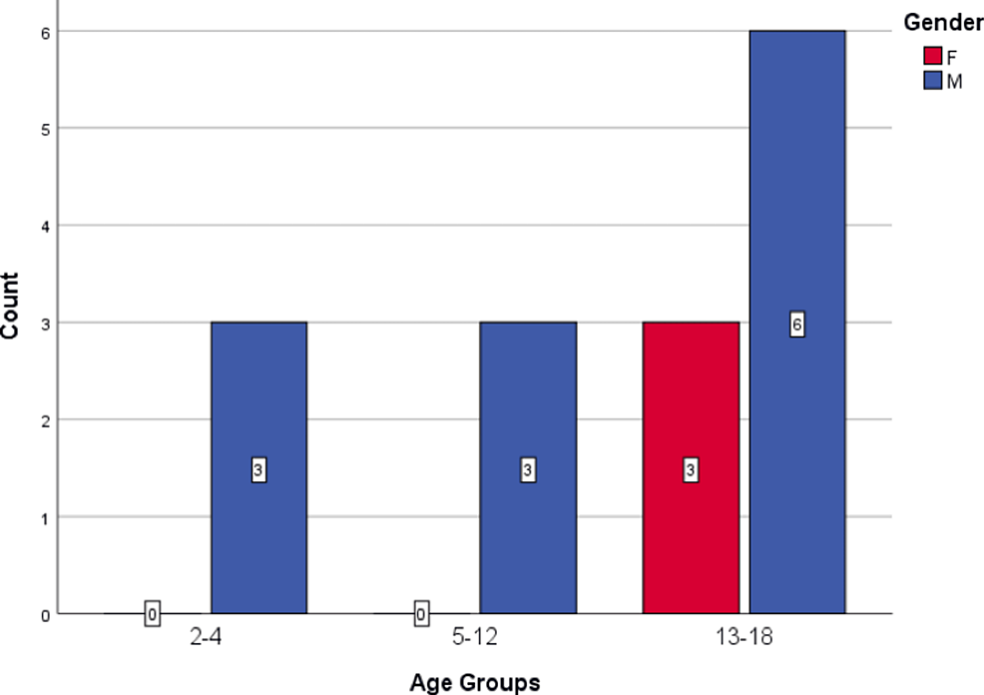
A bar chart of patients' ages with their gender Age groups are presented in years; F: female; M: male

This study included 15 pediatric patients treated at the pediatric department of KAUH from November 2008 to July 2023. Notably, there was a significant male predominance, with 80% (n = 12) being male and 20% (n = 3) female. The median age at the initiation of TPE was 13 years, ranging from four to 18. Figure 1 provides a comprehensive overview of the age distribution categorized by gender.

The mean weight of the study population was 26.4 kg, ranging from 17.7 kg to 75 kg, with an average height of 97 cm, varying from 90 cm to 160 cm.

Approximately 66.7% of patients demonstrated an average severity of illness, measured by the Glasgow Coma Scale (GCS), with a score of 10.7. Additionally, 66.6% displayed an average Expanded Disability Status Scale (EDSS) of 4.8. The median length of hospital stay was 23 days, while the median length of stay in the pediatric intensive care unit (PICU) was 8.5 days.

Primary presenting symptoms among patients included limb weakness (n = 6), loss of consciousness (n = 3), dysphagia (n = 3), photophobia (n = 1), and ascending paralysis quadriplegia (n = 1). Patients underwent TPE for various conditions, encompassing GBS (n = 7) [9], myasthenia gravis (MG) (n = 3), transverse myelitis (TM) (n = 2), neuromyelitis optica (NMO) (n = 2), and systemic lupus erythematosus (SLE) cerebritis (n = 1).

**Figure 2:**
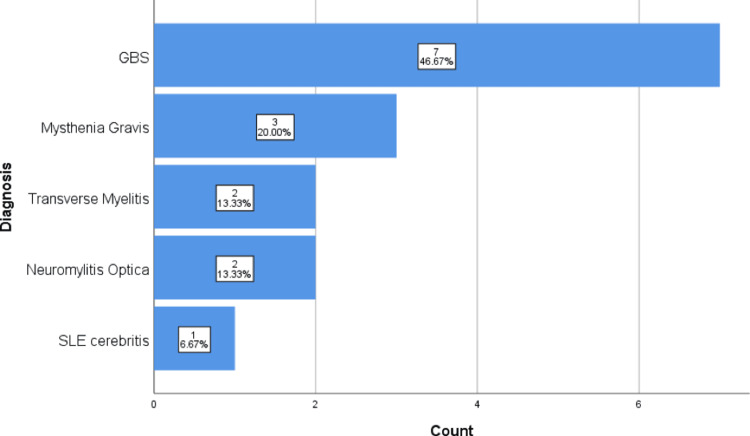
A bar chart of the patients' diagnoses GNS: Guillain-Barré syndrome; SLE: systemic lupus erythematosus

Figure 2 visually illustrates the diagnostic distribution, with 12 patients admitted to the PICU and mechanical ventilation required for 10 patients.

Twelve patients were on steroids; six of them underwent intravenous immunoglobulin (IVIG) treatment, and rituximab was administered to four patients. Notably, among these, IVIG was co-administered with rituximab in one instance, while the remaining cases involved rituximab administration alongside steroid treatment.

In magnetic resonance imaging (MRI) findings, abnormalities were observed in 10 cases, while the remaining five either yielded normal results or did not undergo MRI. Specifically, conditions identified were GBS (n = 5), TM (n = 2), MG (n = 1), NMO (n = 1), and SLE cerebritis (n = 1).

In the subset of three GBS patients who underwent an MRI of the spine with contrast, common findings included increased enhancement and thickening of the nerve root of the cauda equina. Among the remaining two GBS cases, one exhibited multiple tiny, low signal intensity foci in the corpus callosum and periventricular deep white matter, while the other displayed abnormal signal intensities in the left cerebellar hemisphere, periventricular centrum semiovale/corona radiata bilaterally, and the medulla oblongata.

For TM, a combined brain MRI with contrast and an MRI of the cervical spine with contrast in one patient revealed pre-TPE MRI spine T2 hyperintensity involving the cervical, thoracic, and lumbar spine with minimal contrast enhancement. The brain MRI exhibited diffuse leptomeningeal enhancement, and in the post-TPE MRI spine, there was a significant resolution of an abnormal cord signal with minor residual heterogeneity. In the other TM patient, the cervical spine MRI with contrast showed a diffuse spinal cord signal abnormality.

In the case of the MG patient, an MRI of the brain with contrast displayed multiple periventricular deep white matter abnormalities in both cerebral hemispheres and the spinal cord. Patients with NMO exhibited abnormal signals involving the periaqueductal gray matter, mammillary bodies, medulla oblongata (including the area postrema), scattered deep white matter, and juxtacortical T2 hyperintensities on MRI.

**Figure 3:**
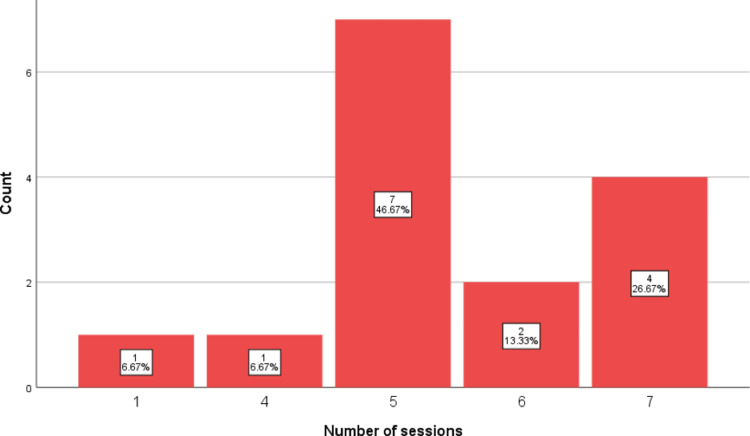
A bar chart of the number of TPE sessions TPE: therapeutic plasma exchange

Lastly, the patient with SLE cerebritis demonstrated parenchymal lesions in bilateral supra and infratentorial regions on an MRI of the brain with contrast and magnetic resonance angiography (MRA) with contrast. As mentioned previously, five patients either did not undergo MRI scans or had results yielding normal findings. Among the four patients who underwent a single MRI scan, abnormal findings were observed. Another patient underwent 10 MRI scans, and resolution was observed one month after TPE compared to one month before it. Additionally, one patient showed disease progression in MRI compared to the previous scan before initiating TPE, while two patients exhibited remarkable improvement in subsequent MRI scans. Figure 3 presents a comprehensive overview of the number of TPE sessions.

Most patients underwent five sessions of TPE (n = 7), with two procedures conducted using the Haemonetics machine and the remaining 13 patients utilizing the Optia machine.

All patients received albumin and normal saline as replacement fluids. One patient had prior apheresis experience, and another patient was undergoing dialysis.

Notably, 12 patients exhibited improvement following TPE (80%), while three patients did not experience improvement (20%). Among these three patients, one demonstrated improvement after a tracheostomy, one faced a disability, and one, unfortunately, passed away, succumbing to their primary condition, refractory status, with no improvement despite TPE. Table 1 summarizes the clinical data and outcomes of our patient cohort.

**Table 1:** A summary of clinical data and outcomes for patients treated with plasma exchange

#	Diagnosis	Related clinical manifestations	Treatments	Outcomes
1	Guillain-Barré syndrome (GBS)	Bilateral lower limb weakness and areflexia	Steroids, intravenous Immunoglobulin (IVIG), heparin, hydrocortisone, ketamine, midazolam, polyethylene glycol, omeprazole, ondansetron, and diphenhydramine.	Recovery of motor function, and ability to walk again, accompanied by a return of reflexes.
2	GBS	Unsteady gait, level of consciousness (LOC), headache, and shortness of breath (SOB)	-	Faced disability
3	GBS	Difficulty in walking, swallowing, and talking, and areflexia	Steroids and IVIG	Increased muscle power, and improved motor function. Reflexes remained absent and swallowing function returned to normal.
4	GBS	Lower limb weakness and absent deep tendon reflexes in the leg	Steroids and IVIG	Demonstrated noticeable improvement in reflex responses.
5	GBS	Ascending paralysis, “quadriplegia”	Amitriptyline, rosuvastatin, enoxaparin, propranolol, risperidone, vancomycin, acyclovir, carbamazepine, and sennosides.	-
6	GBS	Lower limb weakness and decreased deep tendon reflexes in the legs	Steroids and IVIG	Recovery of motor function, and ability to walk again, accompanied by a return of reflexes.
7	GBS	Ataxia, respiratory distress, and areflexia	IVIG	Recovery of motor function, and ability to walk again, accompanied by a return of reflexes.
8	Myasthenia gravis (MG)	Difficulty closing eyes, nasal speech, facial weakness, double vision, and dysphagia	Steroids, pyridostigmine, hydrocortisone, and diphenhydramine	Improvement in nasal speech. This enhancement occurred without observable impairment in facial sensation, although no improvement was noted in eye closure.
9	MG	Difficulty swallowing and talking, dysarthria, and dysphagia	Steroids	Improvement marked by positive gag and cough reflex response, and return of normal swallowing function.
10	MG	Diplopia “double vision”, difficulty swallowing and talking, upper and lower limbs weakness proximal more than distal	Steroids, IVIG, rituximab, pyridostigmine, and azathioprine	Bilateral equal reactive pupils were observed. Despite an enhancement in the gag and cough reflex, swallowing difficulties persisted.
11	Transverse myelitis (TM)	Inability to stand or sit unaided due to lower limb weakness	Steroids, rituximab, pregabalin, enoxaparin, and amitriptyline	Improvement in lower limb weakness and unaided sitting was achieved. standing capability did not show improvement.
12	TM	Hyperesthesia in the lower limb, severely hindering walking ability, and difficulty with voiding	Steroids, enoxaparin, baclofen, and amitriptyline	Enabled the performance of hip adduction and abduction.
13	Neuromyelitis Optica (NMO)	Bulbar symptoms, generalized weakness, cough, dysphagia, constipation, and nausea	Steroids	Demonstrated improvement after a tracheostomy
14	NMO	Cyanosed with LOC, bradycardia, and fixed dilated pupils	Steroids and rituximab	-
15	Systemic lupus erythematosus (SLE) cerebritis	LOC, seizures, and photophobia	Steroids, rituximab, cyclophosphamide, levetiracetam, and phenytoin	Passed away

Regarding complications observed during and after TPE, patients undergoing the procedure experienced various reversible complications, including fever (n = 5), electrolyte disturbance (n = 5), hypotension (n = 3), hypocalcemia (n = 2), bradycardia (n = 2), vomiting (n = 1), tachycardia (n = 1), eye rash (n = 1), infection (n = 1), and bleeding from the central line insertion site (n = 1).

## Discussion

This study focused on a cohort of 15 patients treated at the pediatric department of KAUH between November 2008 and July 2023, specifically examining the most prevalent indications, complications, and outcomes of pediatric patients undergoing TPE at KAUH. Our findings shed light on the clinical profiles and outcomes of TPE therapy in our study population, emphasizing the necessity for personalized treatment approaches and rigorous management of potential complications.

Therapeutic plasma exchange serves as an extracorporeal blood purification technique used in treating various medical conditions [2, 6]. This process involves separating plasma from whole blood and replacing it with either plasma or albumin to eliminate undesirable plasma components. The method facilitates the removal of harmful substances such as antibodies, immune complexes, cytokines, and endotoxins while simultaneously replenishing any deficient plasma components [10].

Considering the updated ASFA guidelines and recommendations, the literature consistently reflects a global consensus on the therapeutic advantages of TPE for various neurological conditions [11]. Sık et al. (2020) extensively documented TPE indications, encompassing autoimmune diseases, neurological disorders, and hematological conditions [12]. Therapeutic plasma exchange is applicable to treat autoimmune diseases like acute disseminated encephalomyelitis (ADEM), anti-N-methyl-d-aspartate (NMDA) receptor encephalitis, and SLE. Hematological conditions, including hemolytic uremic syndrome (HUS), thrombotic microangiopathy (TMA), and hemophagocytic lymphohistiocytosis, also benefit from TPE. Furthermore, TPE finds utility in acute hepatic failure, acute rejection post-liver transplantation, and specific poisoning cases.

Therapeutic plasma exchange holds a pivotal role as a first-line treatment alongside steroids and IVIG for numerous pediatric neurology conditions. Category I neurologic indications for TPE, in accordance with ASFA 2023 guidelines, are summarized in Table 2 [13].

**Table 2:** Category I neurologic indications for therapeutic plasma exchange (TPE) In accordance with American Society for Apheresis (ASFA) 2023 guidelines [13] Ig: immunoglobulin

Condition	Rationale for TPE
Acute inflammatory demyelinating polyradiculoneuropathy	Primary treatment
Chronic inflammatory demyelinating polyradiculoneuropathy	
Chronic acquired demyelinating polyneuropathy	IgG/IgA/IgM related
Myasthenia gravis	Acute, short-term treatment
N-methyl-D-aspartate receptor antibody encephalitis	

We assessed the impact of TPE on 10 patients exhibiting diverse neurological symptoms, revealing varying degrees of improvement. Notably, TPE facilitated the recovery of motor function and restored walking ability in cases of bilateral lower limb weakness. However, the return of reflexes differed among these patients. Furthermore, TPE enhanced muscle power in a patient with bilateral lower limb weakness and difficulty swallowing, restoring normal swallowing function despite persistent reflex absence. In another instance of bilateral lower limb weakness, TPE improved strength for sitting unaided, yet standing capability remained unchanged. Patients with an unsteady gait and areflexia showed temporary improvement in reflex responses post-TPE. Similarly, a patient with lower limb hyperesthesia experienced improved limb function, with toe movement remaining unaffected.

Therapeutic plasma exchange also significantly improved symptoms in patients with difficulty swallowing, talking, nasal speech, facial weakness, and double vision. However, it's important to note that while some improvements were observed, certain symptoms persisted. These findings underscore the potential of TPE to ameliorate a variety of neurological symptoms, acknowledging that the extent of improvement varies based on the specific condition.

Our results align with Hindawi et al. (2023), demonstrating positive responses in MG patients to TPE, positioning it as a valuable option for disease management and potentially reducing the need for ventilator support [8]. We observed enhanced clinical parameters and symptom relief post-TPE, consistent with findings reported by Cortina et al. (2018) and Sık et al. (2020), who also highlighted TPE complications [1, 12]. During plasma exchange, complications may arise, encompassing issues like catheter blockage, circuit clotting, vascular malfunction, allergic reactions, low ionized calcium, rash, bradycardia, hypotension, nausea, vomiting, and dizziness [4]. Typically, these complications are linked to vascular access, the replacement solution, and the overall procedure [14-15]. These findings propose TPE as a valuable therapeutic intervention in this patient population, warranting further investigation and consideration in clinical practice.

A primary limitation of our study is the relatively small sample size, with only 15 patients. Consequently, our findings may lack generalizability to a broader population. A larger sample would offer a representative dataset, enabling more reliable statistical analyses and potentially revealing additional insights into TPE outcomes in pediatric patients. Larger studies could also validate the trends observed in our study, establishing a stronger foundation for clinical recommendations.

Future TPE research should prioritize developing standardized treatment protocols tailored to specific medical conditions and exploring strategies for mitigating treatment-related complications. Special attention to the pediatric population, including age-specific considerations, is crucial.

Long-term follow-up research is essential for assessing the durability of treatment effects, and comparative effectiveness trials should evaluate TPE against alternative therapies. Furthermore, investigating TPE's impact on patient quality of life, economic considerations, underlying mechanisms, and emerging indications will contribute to its optimization and broader clinical utility. These research directions aim to enhance our understanding of TPE, leading to improved patient outcomes and expanded applications in diverse medical scenarios.

## Conclusions

Multiple treatment options are available for children with neuroinflammatory diseases, including plasma exchange (PLEX) therapy. Our center's experience with these diseases and PLEX therapy does not allow us to draw firm conclusions. However, from our observations, we have noticed improvement in some cases, along with a low rate of mortality and morbidity associated with this treatment option. Concerted efforts in conducting prospective studies are needed to fully understand the effectiveness of this therapeutic approach and its optimal use.
